# Connecting process models to response times through Bayesian hierarchical regression analysis

**DOI:** 10.3758/s13428-024-02400-9

**Published:** 2024-05-15

**Authors:** Thea Behrens, Adrian Kühn, Frank Jäkel

**Affiliations:** 1 Institute of Psychology, Technical University of Darmstadt, Darmstadt, Germany; 2 Centre for Cognitive Science, Technical University of Darmstadt, Darmstadt, Germany

**Keywords:** Response times, Cognitive modeling, Hierarchical Bayesian models, Elementary information processes

## Abstract

Process models specify a series of mental operations necessary to complete a task. We demonstrate how to use process models to analyze response-time data and obtain parameter estimates that have a clear psychological interpretation. A prerequisite for our analysis is a process model that generates a count of elementary information processing steps (EIP steps) for each trial of an experiment. We can estimate the duration of an EIP step by assuming that every EIP step is of random duration, modeled as draws from a gamma distribution. A natural effect of summing several random EIP steps is that the expected spread of the overall response time increases with a higher EIP step count. With modern probabilistic programming tools, it becomes relatively easy to fit Bayesian hierarchical models to data and thus estimate the duration of a step for each individual participant. We present two examples in this paper: The first example is children’s performance on simple addition tasks, where the response time is often well predicted by the smaller of the two addends. The second example is response times in a Sudoku task. Here, the process model contains some random decisions and the EIP step count thus becomes latent. We show how our EIP regression model can be extended to such a case. We believe this approach can be used to bridge the gap between classical cognitive modeling and statistical inference and will be easily applicable to many use cases.

Models of mental processes have a long tradition in cognitive science and psychology. They specify a series of (mental) operations necessary to complete a task and can make predictions about the difficulty and successes of different strategies under varying conditions. The amount of mental processing required to complete a task is assumed to be reflected in the time it takes to do so. Response times are easy to measure in psychological experiments and, if analyzed with the right tools, can be very informative about the underlying mental processes. Mathematical psychologists have analyzed the precise theoretical conditions that make it possible to infer mental processes from response time data and found that strong inferences are usually not possible (Luce, 1991; Townsend & Ashby, 1983). In general, it is surprisingly hard to even distinguish between plausible serial and parallel processing architectures. However, there are a few prominent exceptions that allow for strong inferences about the information processing architecture, such as the Wheatstone bridge (Schweickert, 1980) and systems factorial technology (Townsend & Nozawa, 1995). These theoretical insights have been applied successfully to elementary detection, recognition, and search paradigms but had only little impact on applied research, for example, in human–computer interaction where tasks are more complex.

Ultimately, our goal is to fit models for complex cognitive tasks to response time data. In this regard, we are inspired by research in human–computer interaction, where detailed process models have long been used to model real-world reaction times (John & Newell, 1989), albeit without an accompanying statistical analysis.

For example, GOMS models (goals, operators, methods, selection rules) are used to analyze the complexity of a task for a specific user interface. They can make predictions about the time required to complete a task as well as the working memory load for the user (Estes, 2021). They do so by using an inventory of basic building blocks, simple atomic actions, for which duration and memory load have been measured carefully. When building a model for a new task, these building blocks can be combined to get an estimate of the overall duration. Although in this approach process models predict response times directly, they are not adequate as psychological models, as they make predictions about a standard expert user only and cannot be used to fit data and find out about a specific person’s abilities, for example. Similar caveats hold for cognitive architectures, like Soar (Laird, 2019), ACT-R (Anderson, 2007) or Clarion (Sun, 2016).

The modeling approach we will introduce here, however, builds upon simpler and more abstract process models. Process models for which we can count *elementary information processing steps* (EIP steps) for single experimental trials. Such process models have, for example, been used extensively in the decision-making literature (Bettman et al., 1990; Payne & Bettman, 2004; Payne et al., 1988). The duration of a single elementary information process can be estimated by simple analysis tools, such as linear regression. The analysis we advocate for builds on this idea but improves it in several important ways. First, we make the duration of a single EIP step inherently probabilistic, turning the model into a more plausible cognitive model. For simplicity, however, we will focus solely on serial processing models where each EIP is identically distributed for each participant and there is across-stage independence (Townsend & Ashby, 1983). Second, we use gamma distributions instead of normal distributions to model the duration of an EIP step. Gamma distributions are more adequate for response time models and were already advocated by Maris (1993). They also allow us to analyze the observed response times with a linear gamma regression. In fact, Maris (1993) proposed a general framework for linear gamma regression that is extremely similar to our basic model. Third, because the model is at its core just standard linear regression but with gamma distributions, we can make the model hierarchical and jointly fit the model to the data of individual participants by using standard tools from Bayesian statistics (Gelman & Hill, 2007). Lastly, we add some extensions to the basic model such that it can also deal with situations where the exact EIP step count is latent or there are several strategies with associated process models and EIP step counts. We call the resulting analysis (hierarchical) EIP regression.

Classical examples, where this analysis can be applied, include children’s addition, which can often be well predicted by the smaller of the two addends (Groen & Parkman, 1972), mental rotation, where response times increase linearly with angular difference between the two stimuli (Shepard & Metzler, 1971), and visual search in a feature-conjunction display (Treisman, 1982). In these cases, the regression models are not just mere statistical tools to analyze the data, they can be interpreted as simple cognitive models. The slope of the regression tells us something about the processing speed of some cognitive component. In the case of simple addition, the elementary information process is counting up a number. A regression model then tells us something about the speed of mental counting. When our model predicts half a second increase in response time for each increase in the smaller addend, that means that children need about half a second to carry out one EIP step, i.e., counting up one number. In the classical mental rotation experiment where two depictions of 3D objects had to be compared, the response time increased linearly with the angular difference between the two depictions. The fact that the data could be fit by linear regression, with the angular difference as a predictor of response time, corroborates the hypothesis that the internal process seems to involve mentally rotating one of the objects to match the image of the other. We also learn that this mental rotation seems to be done at approximately constant speed which we can read off of the regression slope.

In this paper, we demonstrate how this big class of process models can be fit to response time data. In all cases where the cognitive process can be expressed in counts of elementary processing steps (EIP steps), they can be fitted with the model we present here. The serial processing model that we assume may, of course, be wrong. EIP steps might not follow a gamma distribution. Even if they do, they might not follow the same distribution within each participant. This could be because there are really several different elementary processes instead of just one, or the speed of processing at one stage might depend on the speed of processing at other (earlier) stages and, therefore, across-stage independence does not hold. In general, the processing might also happen (partially) in parallel. As mentioned before, it is surprisingly hard to identify the true serial or parallel architecture from response times alone (Townsend & Ashby, 1983). However, for many practical applications – e.g., in human–computer interaction or as in the above example, where children count on their fingers – these assumptions are plausible, and the true architecture is of less interest than a model of the expected increase in response time with relevant task variables.

## EIP regression

We assume that each EIP step takes some time, so that trials that require more EIP steps will, on average, have proportionally longer response times than trials with fewer EIP steps. Additionally, there is a constant offset component in the model, accounting for processes that are the same in all trials (e.g., orienting oneself or initiating the motor response of pressing a key). Hence, the expected response time of participant *i* on trial *j* can be described with the following linear equation:

1 $$\begin{aligned} \mu _{ij} = a_i x_{ij} + b_i. \end{aligned}$$

This mean response time $\mu _{ij}$ is given by the constant time $b_i$ (accounting for the steps that are the same across trials) and the parameter $a_i$ that is multiplied by the number of EIP steps $x_{ij}$ (that vary between trials). We want to estimate the two parameters $a_i$ and $b_i$ to find out the processing speeds for each participant.

**Fig. 1:**
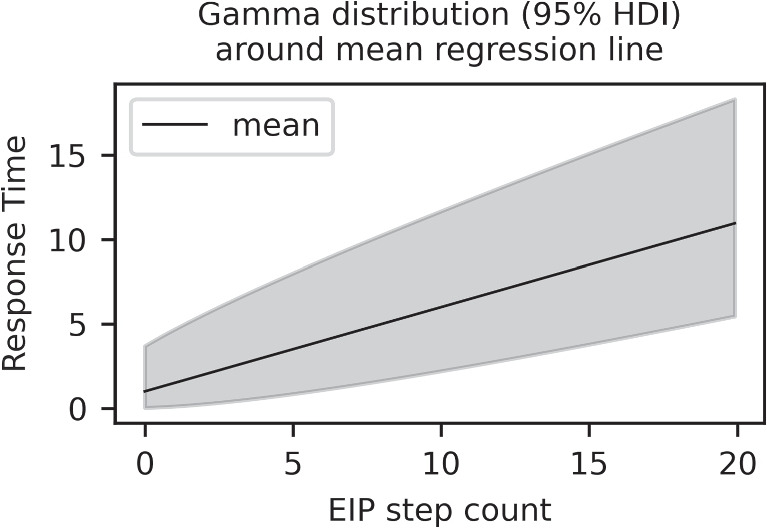
Regression line with asymmetrical gamma density around it. Here the parameters are $a=0.5$, $b=1$, $\theta =1$. With an increasing count of EIP steps, the spread increases

In linear regression, a noise term – usually normally distributed with constant variance – is added to the mean to account for the variability of the data. Here, instead, we assume that every single EIP step is of random duration. EIP steps will not take the exact same amount of time in each execution. We model each EIP step as a draw from a gamma distribution with mean $a_i$ and the constant part as a single draw from a different gamma distribution with mean $b_i$. The fact that gamma distributions are skewed and only defined on positive values make them very well suited for modeling the duration of an EIP step (Maris, 1993). A natural effect of summing several random EIP steps is that the expected spread of the overall response time increases with a higher EIP step count (see Fig. 1 for an example of how the regression line with the gamma density around it might look like). Hence, using gamma distributions for EIP steps fixes two conceptual flaws compared to simply assuming normal errors and homoscedacity for Eq. 1, as it is usually done. First, response times are constrained to be positive and, second, the variance increases with the step count, as it should.

Let $z_n \sim \text {Gamma}(k_n, \theta _i)$ be the random processing time of the $n^{\textrm{th}}$ EIP step in one trial of participant *i*. Usually, gamma distributions are specified using parameters for shape, *k*, and scale, $\theta$. Mean and standard deviation of the distribution are then $m = k \theta$ and $s = \sqrt{m \theta }$. We prefer, however, to parameterize the gamma distribution directly by mean and standard deviation because they are easier to interpret. We denote this parameterization of the gamma distribution Gamma ^′^. With $m_n=k_n\theta _i$ the random processing time $z_n$ for the $n^{\textrm{th}}$ EIP step can then equivalently be written as

2 $$\begin{aligned} z_n \sim \text {Gamma}'(m_n, \sqrt{m_n \theta _i}). \end{aligned}$$

We assume that the $z_n$ are identically and independently distributed. Psychologically, this means that for each participant there is just one elementary information process and that there is across-stage independence (Townsend & Ashby, 1983).

We also assume that the EIP steps are processed serially, hence, we now want to know the total response time distribution of $N+1$ random steps, $y=\sum _{n=0}^{N} z_n$. We assume all gamma distributions of participant *i* share the same scale parameter $\theta _i$. In this way, the overall response time distribution for *y* is easy to compute because the sum of several independent gamma variates $z_n$ from distributions with the same scale $\theta _i$ and shape parameters $k_n$ is again gamma distributed: $y \sim \text {Gamma}(\sum _{n=0}^{N}k_n, \theta _i )$. The mean of this gamma distribution is $M = \theta _i \sum _{n=0}^{N} k_n = \sum _{n=0}^{N} m_n$ and its standard deviation is $S = \sqrt{M \theta _i}$, and, hence, the overall response time $y \sim \text {Gamma}'\left( M, S \right)$ in our alternative parameterization of the gamma distribution.

Remember that each participant *i* is modeled by three parameters that describe the gamma-distributed response time of each trial: The mean time each EIP step takes, $a_i$, the mean offset, $b_i$, and the scale of the gamma distributions $\theta _i$. Hence, if participant *i* needs $N=x_{ij}$ EIP steps on trial *j*, $m_0=b_i$ for the initial step and $m_n=a_i$ for the other identical EIP steps (where *n* ranges from 1 to $x_{ij}$), then the overall mean of the summed EIP step times is $M=a_i x_{ij} + b_i = \mu _{ij}$ (1) with standard deviation $S = \sqrt{\mu _{ij}\theta _i} = \sigma _{ij}$. The random response time $y_{ij}$ of participant *i* on trial *j* is therefore

3 $$\begin{aligned} y_{ij}=\sum _{n=1}^{x_{ij}} z_n \sim \text {Gamma}'\left( \mu _{ij}, \sigma _{ij} \right) . \end{aligned}$$

The basic EIP regression model is thus a linear gamma regression with the intuitive interpretation of a latent random duration for each processing step. Maris (1993) proposed an extremely similar but much more general linear gamma regression for response time data where the shape and the rate (not the scale) parameter of a gamma distribution are linear functions of observable predictors. In our model, the mean $\mu _{ij}=a_i x_{ij} + b_i$ (1) and the variance $\sigma _{ij}^2=\mu _{ij}\theta _i$ are tied together and are linear functions of the number of EIP steps $x_{ij}$. While Maris (1993) prominently notes the connection of his model with serial processing models and applies his model to mental rotation data, he does not derive his gamma regression directly from adding latent random processing steps.

Importantly, because we know the distribution of the sum of the latent gamma variates, we do not have to model the duration of the single EIP steps $z_n$ explicitly. This is a great computational advantage in the hierarchical Bayesian model that we propose in the next section. Other commonly used distributions for response times (e.g., the Weibull or log-normal distributions) do not have this property and would therefore be a lot more cumbersome to work with.

In later sections, we will extend the use of the EIP regression model to cases where not just the duration of the intermediate steps is unknown but the overall number of EIP steps is also latent. If we assume a distribution for the number of steps, the expected response time of participant *i* on trial *j* will be

4 $$\begin{aligned} \begin{aligned} \mathbb {E}(y_{ij} \mid a_i, b_i, \theta _i)&= \mathbb {E}(\mu _{ij} \mid a_i, b_i, \theta _i) \\&= \mathbb {E}(a_i x_{ij} + b_i) \\&= a_i \mathbb {E}(x_{ij}) + b_i. \end{aligned} \end{aligned}$$

As we know the distribution of $x_{ij}$, we can calculate its expected value. The parameters $a_i$ and $b_i$ for each participant can therefore be identified from the mean response times if there are at least two experimental conditions with different expectations for $\mathbb {E}(x_{ij})$.

While it is possible to estimate the parameters from the means, it is statistically much better to try and model the full response time distributions $y_{ij}$ to infer the parameters. In order to do so, we need to take into account the full distribution of the number of EIP steps $x_{ij}$. This complicates the statistical modeling considerably because, in this case, the response time distribution is a mixture of gamma distributions. Before we introduce these complications with an example model for response times in a Sudoku task, we will first present a hierarchical extension to model individual differences and apply this model to response time data of children adding numbers.

### Bayesian hierarchical EIP regression

By adding one layer of priors, we can extend the basic gamma regression model to a hierarchical model and estimate the parameters of several participants in parallel. Hierarchical Bayesian models have big advantages over estimating each participant on its own: parameter estimation becomes more robust and one can borrow strength across participants (Rouder et al., 2003; Gelman & Hill, 2007).

**Fig. 2:**
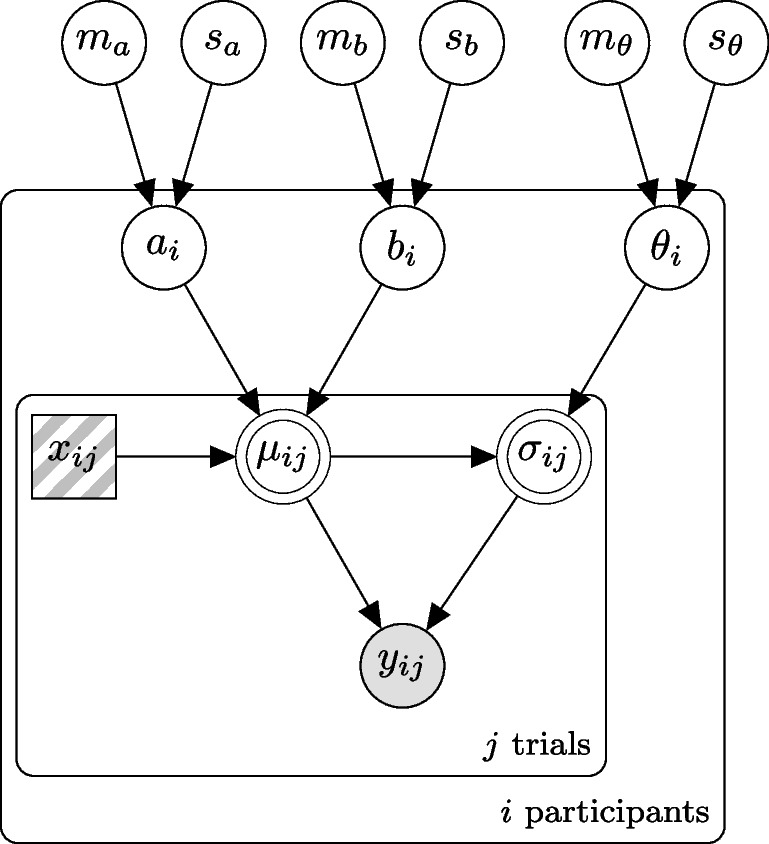
Hierarchical EIP regression model. The *outer box* is the core regression model for each participant *i*. The observed response time $y_{ij}$ depends on a known (or latent, that’s why it’s hatched) number of EIP steps $x_{ij}$. The expected response time $\mu _{ij}$ is a linear function of the number of steps with slope $a_i$ and offset $b_i$. The standard deviation $\sigma _{ij}$ scales with $\theta _i$

As the parameters for each participant ($a_i$, $b_i$, and $\theta _i$) all need to be positive, we sample them from log-normal priors. Usually, log-normal distributions are characterized using parameters $\mu$ and $\sigma$, which are the mean and standard deviation of the logarithm of the distribution (which is a normal distribution). Similar to the gamma distribution, we define a different parameterization denoted by LogNormal ^′^, using the mean and variance of the distribution itself. This parameterization leads the parameters to be measured in seconds and, hence, makes them easier to relate directly to the data. Both of these hyperparameters need to be positive, too. For the hyperpriors on the means, we use a half-normal distribution with $\sigma = 5$. For the hyperpriors on the variances, we use half-Cauchy distributions with $\beta = 5$. These values proved to be reasonable in our applications of the model. They do favor small values but are wide enough to allow for a wide range of values.

The graphical model of our hierarchical EIP regression can be found in Fig. 2. In the display of the graphical models we follow, the conventions used in Lee and Wagenmakers (2014): Round nodes are continuous, squared nodes represent discrete values, open nodes are latent variables, shaded ones are observed; a node with double border is deterministic. In addition, plates enclose parts of a graph to denote independent replications.

This basic model can be extended to more complex cases when the number of EIP steps for a trial are not observed or when there are several tactics to solve a task which would produce a different number of EIP steps. We will deal with such extensions later in the paper. In general, we implemented all graphical models discussed in this paper with PyMC (Wiecki et al., 2022; Salvatier et al., 2016) and used the No-U-Turn Sampler (NUTS, Hoffman & Gelman (2014)) to find good parameter values. We let four chains run in parallel for 2000 iterations, after tuning the hyperparameters of NUTS with 1000 samples which were discarded. Convergence was checked via visual inspection of the traces as well as the diagnostic parameters $\hat{R}$ and effective sample size (ESS) (Vehtari et al., 2021). In Appendix 1 we present the results of a parameter recovery study where we show that, in this way, the parameters of the model can indeed be recovered as expected. Furthermore, we show for one of the experimental designs that we use below, how the posterior density intervals for the parameters depend on the number of participants and the number of trials for each participant. The model parameters can be recovered reasonably well with the amount of data that we have. The code for all models can be found here: https://osf.io/rgh3j/.

## A first example: Addition

The simplest possible use case for the model is when there is just one cognitive tactic that we want to model and the EIP step count for each trial is known. We will show the application of the model in one such simple case: Adding two numbers.

When children learn to add, they usually start by putting up their fingers for each addend and then simply count the fingers (Siegler & Jenkins, 1989). Before they reach the proficiency level of adults and can retrieve the answer to small addition problems from memory, they usually discover several shortcuts to counting explicitly through all the numbers from one to the sum. A quite sophisticated tactic, called min-counting, is to start counting at the larger of the two addends (e.g., $3 + 5$: start at 5 and count three numbers up to get the answer) (Siegler & Jenkins, 1989). During the learning process, children usually use several tactics concurrently. With experience, the most efficient tactics come to dominate, which are the retrieval tactic followed by the min-counting tactic (Siegler, 1987).

Hopkins and Bayliss (2017) examined what tactics children in 7th grade use to solve simple addition problems where both addends are single digits. Here, we use their data to illustrate how our model can be used to infer the temporal properties of EIP steps in the min-counting tactic. Two hundred children from 13 schools with a mean age of 12.38 years took part in the study. The addition part of the study consisted of 36 trials, all single-digit additions with addends greater than 1. After each answer a child gave, the experimenter asked how they arrived at the answer. Answers were classified as min-counting, retrieval, decomposition, and other (e.g., “don’t know”). For details of the experiment, see Hopkins and Bayliss (2017). Here we are only concerned with the min-counting trials which make up about a quarter of the data (of the 6855 correct trials, 1786 were min-counting trials).

The process model for min-counting trials comprises the following parts: read the question, find the bigger number, count from that number as many steps up as the other number indicates, state the answer. Each counting step in this model thus constitutes an EIP step. We can therefore map the counting speed of child *i* onto the parameter $a_i$ and the duration of the other processes onto the parameter $b_i$.

### Results of EIP regression

The estimates for the group parameters of the Bayesian hierarchical EIP regression model can be found in Table 1. These group parameters tell us the mean parameter values and how the parameters vary over participants. For example, looking at the mean values for *a* and *b*, we see that, on average, the offset parameter is roughly four times as big as the slope parameter. Hence, on average, the constant processes (orienting, response initiation, etc.) take about four times as long as each counting step.

**Table 1:** The parameters found for the EIP regression for the counting trials

	Mean	SD	HDI 2.5%	HDI 97.5%
$$m_a [s] $$	0.434	0.029	0.379	0.493
$$m_b [s] $$	1.724	0.084	1.561	1.889
$m_{\theta } [s]$	0.509	0.047	0.421	0.602
$$s_a [s] $$	0.293	0.040	0.221	0.373
$$s_b [s] $$	0.447	0.100	0.253	0.637
$s_{\theta } [s]$	0.485	0.089	0.324	0.653

The *m* parameters are the mean and the *s* parameters the standard deviations of the prior distributions of the respective participant parameters. All values are in seconds

**Fig. 3:**
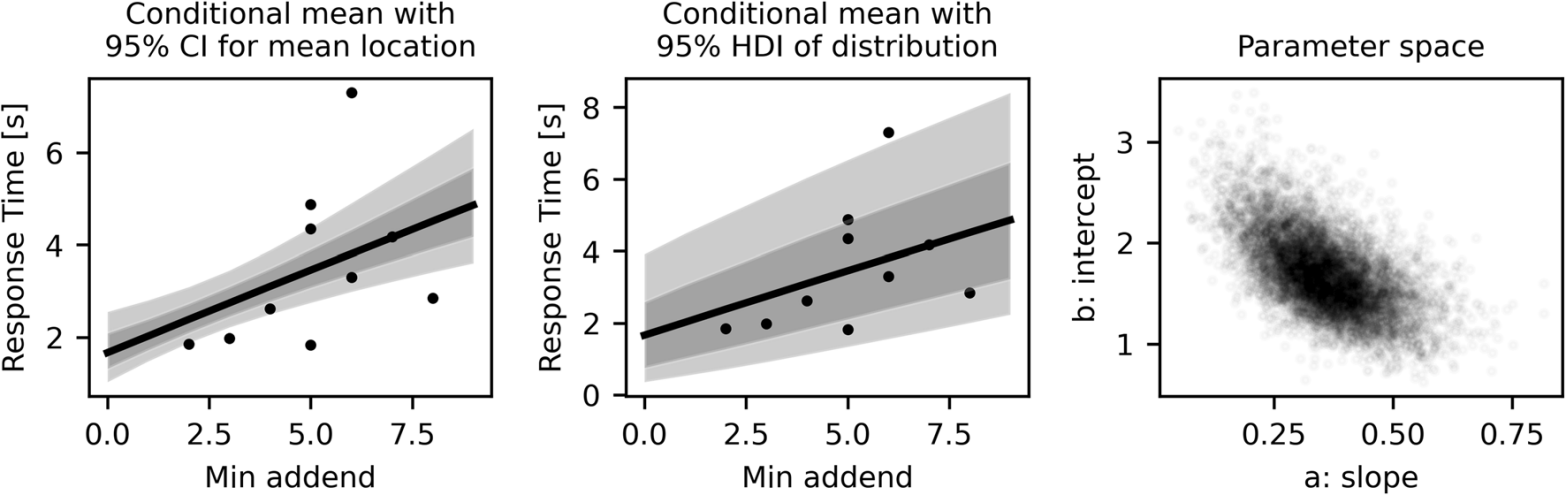
Results for one exemplary participant for the EIP model of min-counting. The regression line and the data points are the same in the first two plots. In the right most plot the individual samples of the two parameters *a* and *b* are depicted

**Fig. 4:**
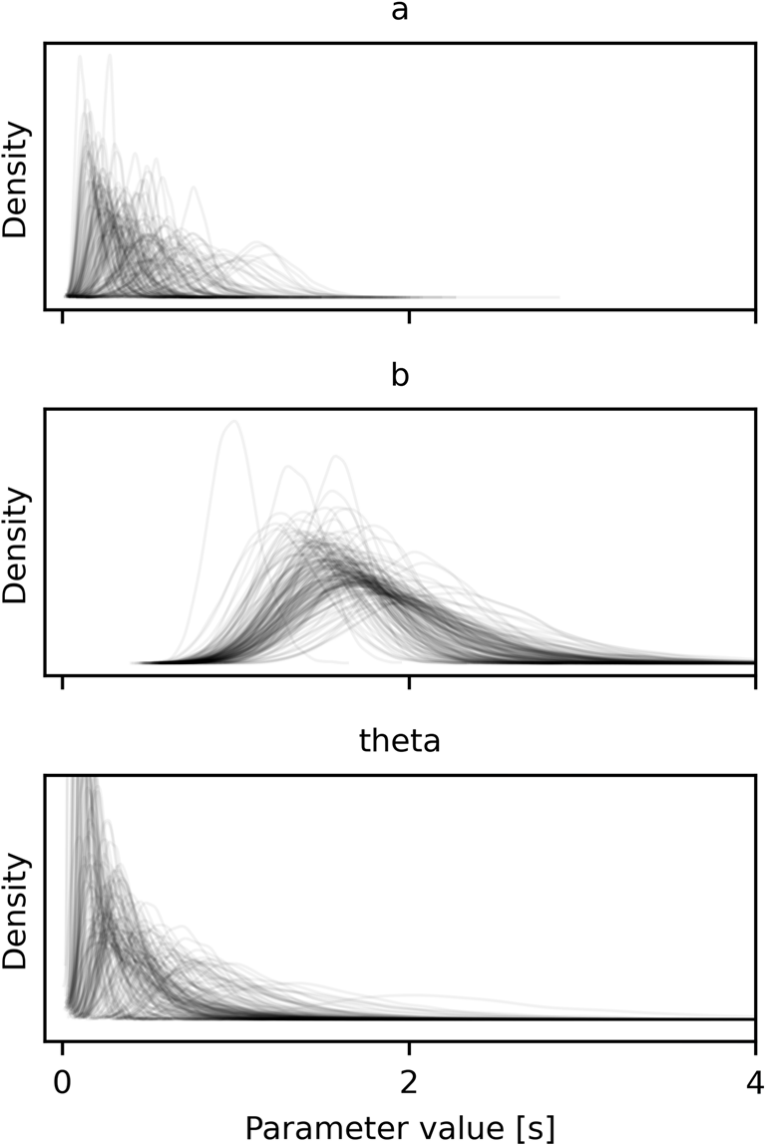
The distributions of the participant parameters for all participants for the EIP model of min-counting. Each line shows the posterior density of values for the respective variable for one participant

For each participant, we also get the individual posterior distributions of all three parameters. The distribution of parameter densities for all participants can be seen in Fig. 4. The regression line for a single, exemplary participant can be seen in Fig. 3. The plot on the left of Fig. 3 shows the conditional fit for the location of the mean (given the data) with its confidence intervals (95% HDI). These are calculated as the respective quantiles of the matrix $a_i x + b_i$, where *x* is the vector [2, ..9] and $a_i$ and $b_i$ are all the posterior samples of the respective values for participant *i*. The *x*-axis depicts the EIP step count, the *y*-axis the response time in seconds. For each participant, we also get the individual posterior distributions of all three parameters. The distribution of parameter densities for all participants can be seen in Fig. 4.

The dots mark individual response times of this participant (only those trials where the participant reported they had used “counting” to find the answer). There were three trials with the minimum value 5 (with response times varying between 1.8 to 4.8 s), for all other minimum values at most two trials were solved by counting by this participant. The solid line is the mean of all posterior regression lines of this participant. Despite the hierarchical model, the 95% HDI around the mean is still on the order of 1 s. However, note that the regression of this student is based on only a few trials. We again see that the constant offset is relatively large, around 2 s, compared to the average time this participant needs to do one counting step. In the middle of Fig. 3, the same mean line is depicted, the shaded area around it illustrates the density of the gamma distribution around it. Except for one data point, all the data are well within the expected variance. To help the reader relate standard linear regression analysis to the EIP regression and appreciate the differences as well as the similarities, we included a linear regression model in Appendix 2. The main results are not much different in the two models; the main difference is the shape of the distribution of the data around the regression line.

## A more complex example with latent steps: Sudoku

In the previous example, the number of processing steps that each participant went through was known. Hence, we could simply plot the number of EIP steps against the response times and the linear fit gave us estimates of the offset and processing time of each EIP. However, for more complex tasks, usually, we will not know the number of processing steps exactly. As an example for such a task, we will look at Sudoku. In general, there are often different ways to solve a problem. In the previous addition example, there was min-counting and retrieval from memory. For Sudoku, too, there are different tactics to fill a cell in a puzzle. Contrary to the data for the addition example, where we knew how a student solved the addition problem, here, we will look at data where we do not necessarily know which tactic was tried.

Furthermore, and also contrary to the counting example, tactics are rarely so simple that each participant executes the tactic in the same way in each situation. Participants do not follow a tactic deterministically but make probabilistic choices. For example, in Sudoku, a participant might sometimes choose to first look at the columns and then at the rows and sometimes do it the other way round. Thus, the number of processing steps needed to solve a problem is not fixed but random and, usually, unobserved. Fortunately, the basic EIP regression model can be extended to deal with these complications (see Fig. 7).

In this section, we will first cover a previously published study on Sudoku (Behrens et al., 2022) to illustrate how these additional complications commonly arise in problem-solving data. In this previous paper, we also published a process model for how people solve Sudokus but only applied it to group data. Here, we will showcase how Bayesian hierarchical EIP regression can be combined with the process model to analyze these data at the single-subject level. These analyses will be very similar for other problem-solving tasks where the results of some reasoning processes can be observed in behavior, but many cognitive steps are latent.

### Sudoku tactics

**Fig. 5:**
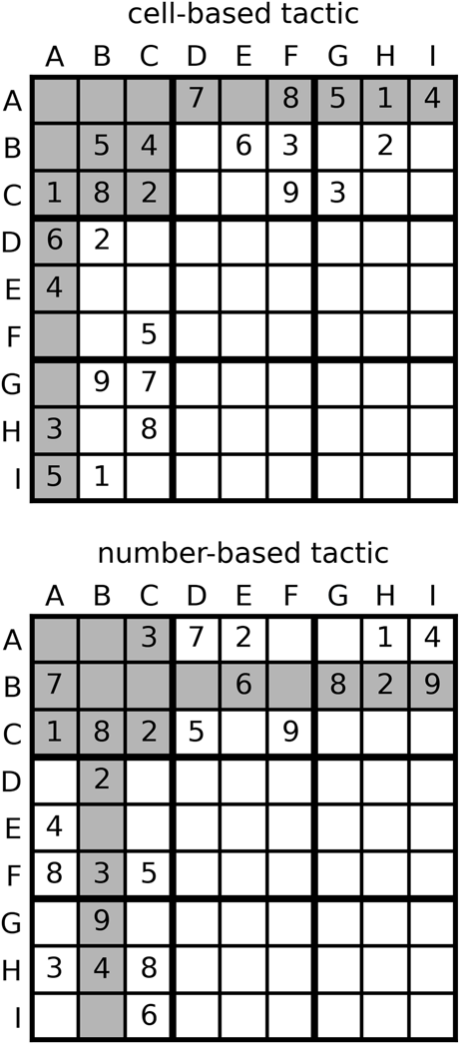
Examples for the two tactics. The correct answer is 9 for the cell AA in both puzzles. For the cell-based tactic, the easiest way to find the answer is by asking “what number can I place in cell AA?” The easiest way for finding the answer in the number-based puzzle is by asking “where in the upper left box (A:C - A:C) can I place the 9?”

Let us first look at the two most simple tactics in Sudoku. They work with different elements as focus: “*What* number can I place *in this cell*?” vs. “*Where* can I place *this number*?” The cell-based tactic (CB for short) tests for a single empty cell of the puzzle, which numbers can be excluded from it by looking at the surrounding cells. When a number appears already in the same row, column, or box, it cannot be placed in the cell under consideration. If all but one number can be excluded, the one remaining number is the solution for the cell. The number-based tactic (NB) focuses on a specific number which occurs already several times on the board. When it does not yet occur in some unit, for example a 3-by-3 box, one can see whether the other occurrences of the number restrict where in the given box this number can be placed. If all empty cells of the box but one can be excluded as locations for the number, the one remaining cell has to be filled with the number (see Fig. 5 for examples of the tactics).

**Fig. 6:**
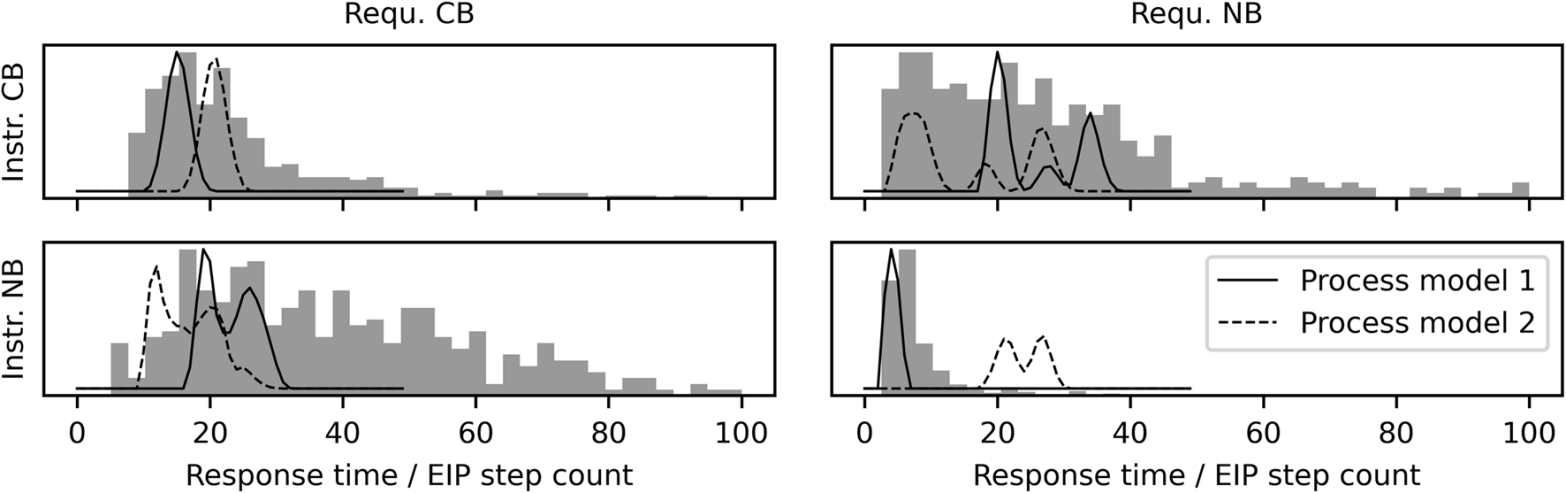
The *grey bars* represent the response times of the participants in the four conditions of the Sudoku experiment and the *lines* depict the EIP step counts for two different strategies. For the first Sudoku model, only the solid lines are relevant. They show the EIP step counts for the process model of the first strategy we implemented. The *dashed lines* (Strategy 2) show the alternative process model that is additionally used in the EIP model with strategy selection. Note that the *x*-axis depicts seconds for the response times but ‘number of EIP steps’ for the scan distributions

### Experiment and instruction groups

In the experiment, we had two different instruction groups that were supposed to bias participants to use one or the other tactic. The task for participants of both instruction groups was to fill in one correct number in a given Sudoku per trial. In order to do so, they had to click on an empty cell with the mouse and then enter a number via the keyboard. In the cell-based instruction group, one cell was highlighted, and the instructions were “Please fill in a number in the highlighted cell”. For the number-based instruction group, a 3-by-3 box was highlighted and the instructions were “Please fill in the X into the highlighted box” (where X was replaced with a specific number in each trial). Both instruction groups saw the same puzzles in a random order, half of which could only be solved with CB tactics, the other half only with NB tactics. Hence, if a participant always started with the same tactic, in half of the cases it led to the correct answer, in the other half they would have to follow up on the first attempt with the second tactic in order to successfully solve the puzzle. The experiment was thus a $2 \times 2$ design with the independent variables instruction and required tactic. The data set, the experimental materials, and the analysis code are available at the OSF project site https://osf.io/rgh3j/. The data set consists of 46 participants, 23 per condition, who met the inclusion criterion (at least 75% correct responses). Every participant completed 36 trials; all saw the same puzzles in randomized order. For both required tactics, we had two seed puzzles and created nine isomorphic stimuli from them. The isomorphs were created by exchanging rows and columns and interchanging the values for different numbers. Although the isomorphs look different on the surface, they afford the same logical inferences and the process model predicts the same number of EIP steps for them.^1^ We, therefore, do not distinguish between the individual puzzles in the following analyses, but just use one distribution of EIP step counts for each combination of instruction and required tactic.

### A first process model

The information given in the two instruction groups is different (fixed cell vs. fixed number) and the process model we developed accordingly also covers the two different cases. The basic processing step that we use in the process model is to search for a number in a given unit (e.g., looking for the 3 in the first row). We call such a step a scan. Scans are the EIP steps for all following models. Note that each scan consists of several sub-steps, i.e., looking at each cell in a unit, that are not modeled explicitly. The model makes predictions about the required number of scans for a given puzzle and instructions. Importantly, the process model makes some random choices and, therefore, the number of EIP steps needed in a trial varies randomly. For example, in Fig. 5 in the CB example, participants will at some point have to scan the A-row and the A-column for a 3 to check whether it can be filled into cell AA. We assume that participants will randomly either first check the A-row or the A-column. Participants who first check the A-column get lucky and only need one scan to exclude the 3. Participants who first look at the A-row will also have to search the A-column and therefore need two scans. In the case where the first executed tactic does not lead to a unique answer, the other tactic has to be applied as a follow-up. The strategy we implemented as a process model here is to always start with the tactic that best fits the instruction they see. We thus expect more scans in incongruent trials, i.e., where the required tactic does not match the one favored by the instruction type. For a detailed description of the proposed algorithms behind the process model, see Behrens et al. (2022). We let the model run on the puzzles for 1000 trials to get a distribution of the EIP step count in each of the four conditions.^2^ Hence, instead of knowing the precise number of EIP steps for each trial as in the min-counting example, here we only know the probability distribution over the number of EIP steps that will be needed by our stochastic process model. In Fig. 6, the solid lines show the distribution of EIP step counts in the different conditions.

### EIP regression with latent steps

The core of the model is identical to the model we used for the counting data. The added complexity stems from the fact that we do not have a definite EIP step count per trial, but a distribution over EIP steps instead. We still want to estimate how long each participant needs on average to carry out an EIP step, i.e., a scan. Additionally, we estimate an intercept term to take care of the processing time not captured in the process model (reading the task, typing the answer, etc.). Of course, this is also a validation attempt of the process model. Only if the model makes reasonable predictions about the required number of EIP steps in the different conditions (or at least their relative proportions) can a good fit be found that explains the response times mainly on the basis of the EIP step count.

As mentioned above, before we fit the EIP regression model, we first let the process model run on the stimuli to get a discrete distribution of the EIP step counts per condition. As we now know this distribution, we can treat the number of EIP steps $x_{ij}$ as a latent variable in our Bayesian model. The only change that is required to the original Bayesian hierarchical EIP regression from before is that we need to provide the discrete prior distribution for each $x_{ij}$ (that depends on the stimulus that was shown in trial *j* and the instruction participant *i* received). Hence, each $y_{ij}$ is now not gamma distributed anymore but a mixture of gamma distributions over the latent number of EIP steps. When fitting the EIP regression model, we could just draw from the latent distribution to get one specific EIP step count for each draw. Instead, we marginalize over this distribution and work directly with the mixture of gamma distributions for $y_{ij}$. Marginalization has the advantage that the samples converge more quickly to a stable distribution. Without marginalization, we need to take more samples in each chain to reach convergence. Once the chains have reached convergence, the results are the same in the two approaches, as one would expect given their equivalence. We report the results from the model with marginalization here.

**Table 2:** The parameters found for the EIP regression with latent EIP step count for the Sudoku data

	Mean	SD	HDI 2.5%	HDI 97.5%
$$m_a [s] $$	1.526	0.386	0.945	2.249
$$m_b [s] $$	7.270	1.694	4.315	10.696
$m_{\theta } [s]$	5.243	0.705	3.960	6.639
$$s_a [s] $$	2.458	1.736	0.746	5.346
$$s_b [s] $$	14.424	7.528	4.266	28.945
$s_{\theta } [s]$	4.542	1.249	2.523	6.972

The *m* parameters are the mean and the *s* parameters the standard deviations of the prior distributions of the respective participant parameters

### Results of latent EIP regression

The values of the group parameters can be seen in Table 2. For each participant, *i*, individual values are sampled for the parameters $a_i$, $b_i$, and $\theta _i$. Their mean is depicted on the left of Fig. 10. According to these fits, participants take between 0.1 and 3 s for one EIP step. The intercept term accounts for all the other processes in the response time. It is below 5 s for about half of the participants. The other half has very variable intercept terms, including values of up to 25 s. Such high intercept terms are a sign of a rather poor process model because in our case the response times ranged from about 10 to about 60 s. If more than half of the time needs to be accounted for by the intercept term instead of the sum of the EIP steps, this means that the distributions of EIP steps do not match participant behavior very well. Luckily, not all is lost, and we can extend the model further to better account for the data.

**Fig. 7:**
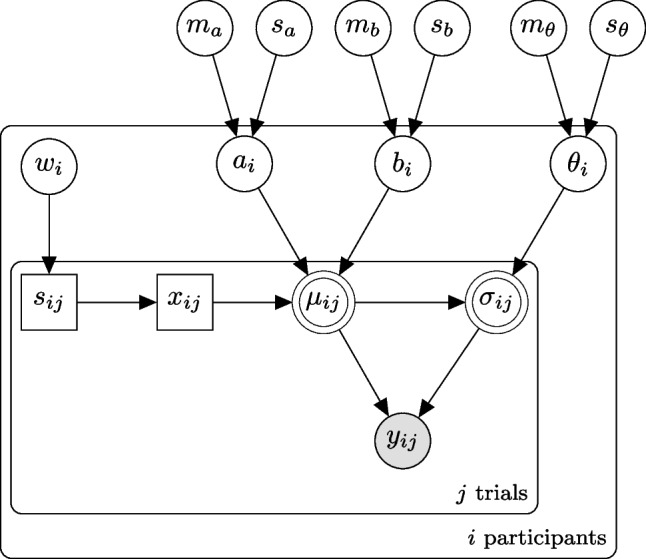
EIP regression model with latent EIP step count and strategy selection for the Sudoku experiment

**Fig. 8:**
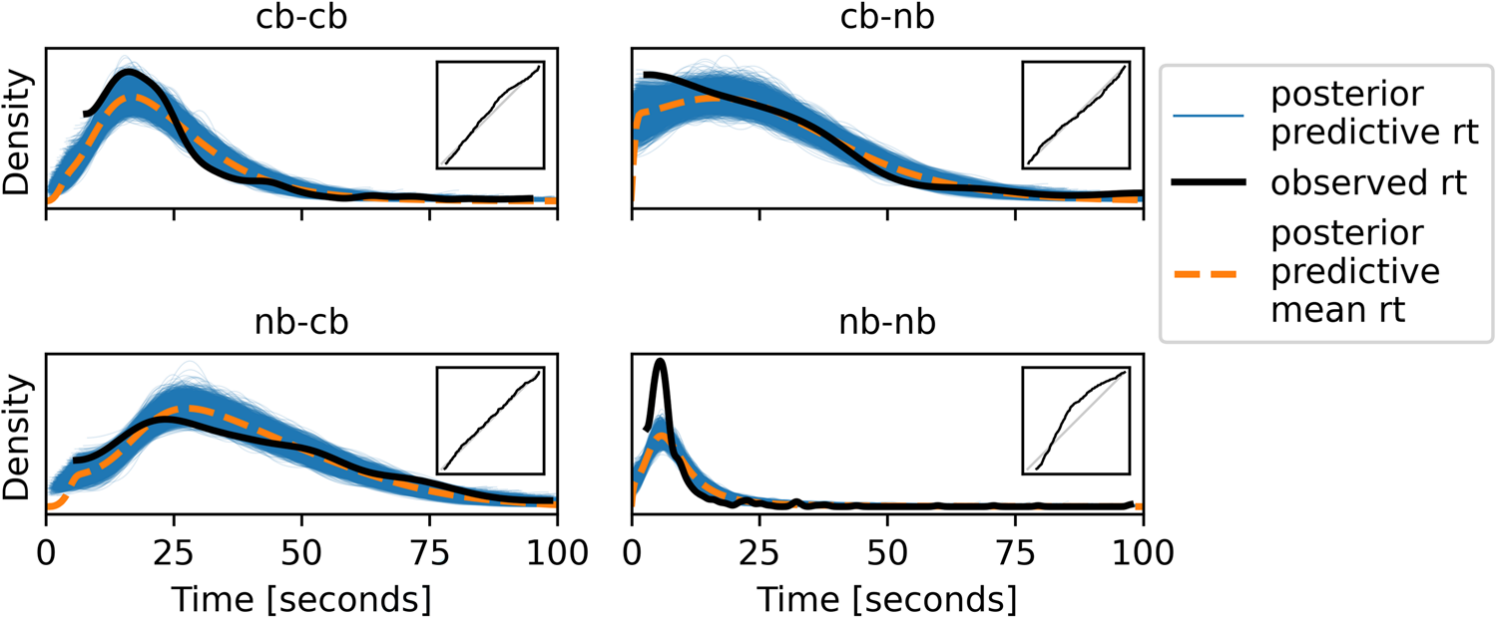
The posterior predictive distributions for the EIP regression model with latent EIP step count and strategy selection for the Sudoku experiment. The inset figures show the PP-plots for the respective condition

### EIP regression with strategy selection

The strategy described above assumes that first the instructed tactic is carried out fully, and only if it did not lead to a unique solution, subsequently the other tactic is carried out. However, it might also be that our participants do not always apply the tactics in this order, instead, they might be starting with the tactic that does not fit the instruction. If they do this, they need more EIP steps in the congruent trials but fewer in incongruent trials. We defined “starting with the other tactic” as a second strategy, implemented a corresponding process model and again sampled the expected numbers of EIP steps per condition to get distributions of scan numbers. The dashed line in Fig. 6 shows the corresponding distributions. We extend the EIP regression model to incorporate strategy selection for each participant. In order to estimate to what extent each of the two strategies explains their response time patterns, for each trial, we draw from a Bernoulli distribution with probability $w_i$ and decide which strategy $s_{ij}$ is used to explain the response time on trial *j*. We sample these weights $w_{i}$ from a uniform beta distribution (Beta(1,1)). From the EIP step distribution corresponding to the selected strategy, one specific EIP step count, $x_{ij}$, is sampled. From here on the rest of the model is the same as in the basic EIP regression model (see Fig. 7 for the corresponding graphical model. Again, we can use marginalization to reach convergence in fewer sampling iterations. In this case, we use the $w_i$ parameter as a mixing weight of the two strategy distributions. Note, however, that the distributions of EIP step counts for the two strategies have considerable overlap (see Fig. 6), so it is probably impossible to say very precisely how much of each of these strategies contributed to the performance of a participant.

### Results of strategy selection analysis

In Fig. 8 the posterior predictive distributions of the model for the four conditions of the experiment can be seen. The lower right part of the figure shows the congruent number-based condition. Here, the observed response times contain many very short answers, which the model cannot match perfectly. The other three conditions are fit very well, as can be seen in the almost perfect diagonal PP-plots in the insets.

**Table 3:** The parameters found for the EIP regression with latent EIP step count and additional strategy selection for the Sudoku data

	Mean	SD	HDI 2.5%	HDI 97.5%
$$m_a [s] $$	1.490	0.141	1.232	1.782
$$m_b [s] $$	2.589	0.950	1.053	4.433
$m_{\theta } [s]$	3.525	0.532	2.557	4.555
$$s_a [s] $$	0.893	0.192	0.577	1.265
$$s_b [s] $$	6.724	10.001	0.548	17.713
$s_{\theta } [s]$	3.296	1.032	1.759	5.344

**Fig. 9:**
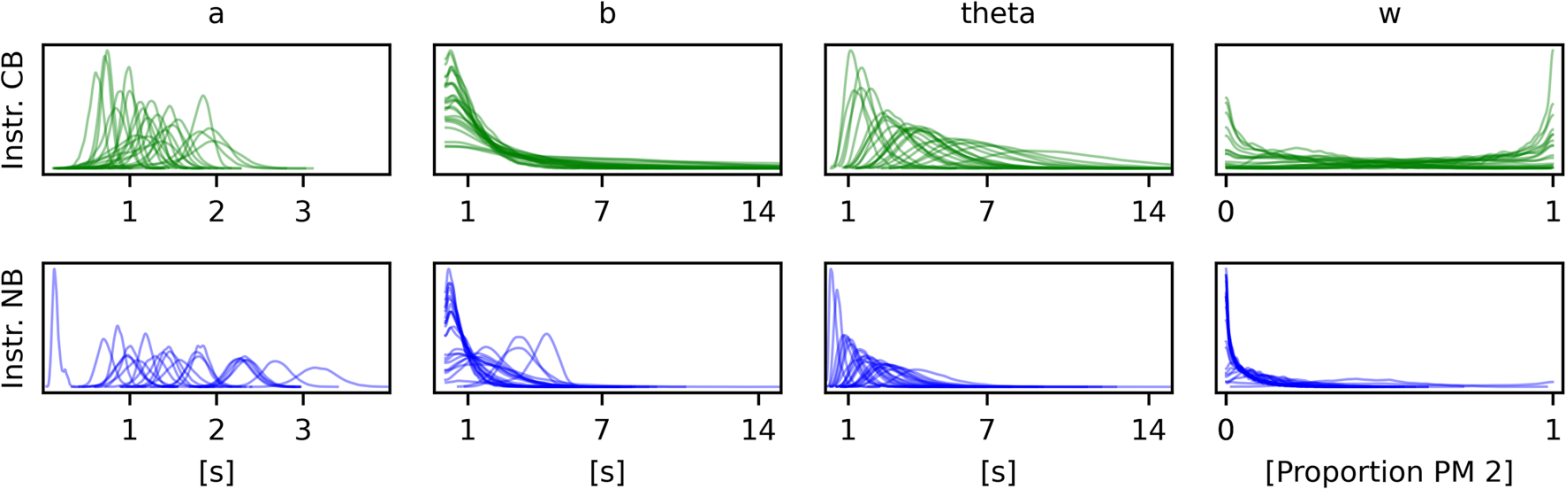
The posterior density of all the participant parameters in the Sudoku model with strategy selection. The two rows of the plot show different instruction groups

**Fig. 10:**
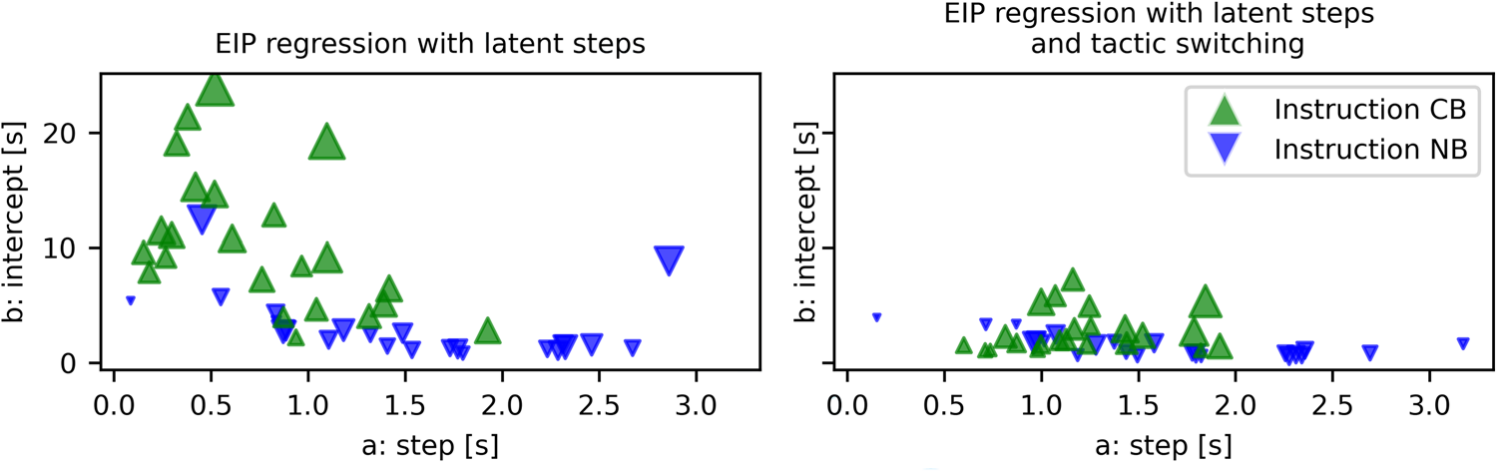
The mean of the $a_i$ and $b_i$ parameters for each participant as fit by the two Sudoku models. The color and orientation of the triangles mark the two instruction groups. The size of the triangles reflects the value of $\theta$, so smaller means better predictable

The values of the group parameters can be seen in Table 3. Compared to the values found by the simpler model, both the intercept term $m_b$ and the variance related term, $m_\theta$, decreased significantly, while slope, $m_a$, stayed relatively constant. The standard deviations of the prior distributions for the three parameters ($s_a$, $s_b$ and $s_\theta$) decreased all at least a bit, indicating that the individual participants now have more similar parameter values compared to the model with just one strategy. The widths of the highest density intervals of all population parameters (except for $s_b$) decreased, showing that the model is more precise in its results. The densities of the participant parameters can be seen in Fig. 9, split into the two instruction groups. The mean of the slope and intercept terms for both models can be seen in Fig. 10. The NB-instruction group gets consistently very low weight parameters for the new strategy (bottom right of Fig. 9), meaning that they overwhelmingly do as the first strategy proposed, i.e., start with the NB-tactic. They were well fit by that model and the additional strategy did not change the fit much. Accordingly, the blue triangles did not move very much between the two plots in Fig. 10. The most striking difference between the two models in Fig. 10 is that the intercept terms, $b_i$, decreased dramatically for many participants in the CB-instruction group. In this group, some participants get a high weight for the newly added strategy. Together, this indicates that around half of the participants in the CB-instruction group did not follow the first strategy’s assumptions, but instead often opted for using the NB-tactic early, even though it was not favored by the instructions. With the additional tactic in the model, a much bigger portion of the overall response time can be explained by the EIP step count assumed in the model instead of needing to be covered by the “catch all” intercept term. This shift alone makes the new model with strategy selection a much better model in our eyes. When the response times can be explained by differences in required EIP steps, it means that there is the possibility of some correspondence between the process models and the processes in the head of the participants.

### Model comparison

Besides the observation that the parameter values are in more plausible ranges in the model with strategy selection as compared to the one without, we can also look at the fit of the data more formally. We use tenfold cross validation to do so. We split the data such that all participants are equally represented in all folds. We train the model on nine tenths of the data and test the performance on the last tenth, which was not used for this training round. This is done ten times, where each fold of the data is the held-out part once. We thus get a measure for how well the model can predict data it has not seen during training. As performance measure we use expected log point-wise predictive density (ELPD) as described by Vehtari et al. (2017), which is the log likelihood of the data given the whole distribution of parameter estimates instead of point estimates of parameters. The ELPD for the EIP regression with latent EIP step count is $-5552.32$ (with a standard error of 49) and for the model with additional strategy selection it is $-5489.08$ (with a standard error of 49), so there is a difference of 63.24 (with a standard error of 18.19 on this difference), showing that the data are much more likely under the more complex model. The ArviZ project (Kumar et al., 2019) also provides a tool to compute an estimated leave-one-out ELPD on the traces. The data contain outlier points resulting in warnings about highly influential observations. Nevertheless, the results by the ArviZ toolbox confirm the results of the cross validation.

## Discussion

We have shown how to connect process models to response time data using gamma regression. The key idea is to treat each elementary information processing (EIP) step as having a gamma-distributed random processing time. We assume the same scale parameter to ensure that the overall response time is also gamma distributed. We call this core model EIP regression. This model can be extended to a Bayesian hierarchical regression model. Such a model allows us to fit the predictor variable of a process model, i.e. the number of EIP steps, to the response time data of individual participants. We illustrated how the model works by applying it to children’s addition (and you can find a comparison to a standard regression analysis in Appendix 2).

In a second example, we developed a process model of a Sudoku task which made predictions about the required number of processing steps in different conditions. As the process model is probabilistic in itself, the predicted number of processing steps is not a single number but a distribution of possible values instead. Even though the number of EIP steps is not observed, the Bayesian hierarchical EIP regression can still be fit to those data. Connecting the discrete predictions of the process model with the quantitative probabilistic model allows us to do two important things: First, we can get an estimate for how long a processing step should take given our process model and the data we collected. Hence, we can make statistical inferences about processes that are never observed in isolation. Second, it also allowed us to statistically assess the process model itself. We saw that our first attempt did not match the data of the cell-based instruction group very well.

The average response time assigned to the processing steps was very short and most of the overall response time had to be explained by the intercept term. We implemented a second process model that allows for starting out with the tactic that does not fit the instruction. In the EIP model, we added a weight parameter for each participant to fit the degree to which each of the two strategies explains the response time patterns. A second version of the process model that allows for starting with different solution tactics improved the fit immensely. The improvement was clearly shown in a model comparison (much better log likelihood of the data) as well as in the values for the individual parameters.

Using a probabilistic programming framework, like PyMC (Salvatier et al., 2016; Wiecki et al., 2022), allows us to define models that would not be expressible in traditional statistical analyses. For example, the generalization from an observed to a latent number of processing steps is straightforward in PyMC but would be very hard using standard regression tools. In general, one motivation for this paper was to showcase how probabilistic programming can be used to bridge the gap between classical cognitive modeling and statistical inference. Traditionally, process models are hypothetical algorithms of how participants solve a problem and make qualitative predictions about processing times, but they are seldom scrutinized statistically. Lee and Wagenmakers (2014) have long advocated the use of Bayesian tools for cognitive modeling and their book provides an excellent collection of basic and advanced models. Our contribution here is to provide one model, EIP regression, that can easily be adapted to many use-cases. If you have a probabilistic process model that you can implement in a computer program, and the program has clearly identifiable elementary information processes, then you can use EIP regression to infer the model’s parameters. Importantly, these parameters have a clear psychological interpretation in terms of the average processing time of an EIP. In addition, as the core model is a simple regression model, the data analysis is very similar to standard analyses in psychology. We thus believe that EIP regression can easily supplement many existing process models.

In fact, decision-making tactics have already been analyzed in depth using EIP models and standard regression tools (Bettman et al., 1990; Payne & Bettman, 2004; Payne et al., 1988). A host of different decision-making tactics exist, some relying on single features, some considering several or all features of the choice options, but they all share the same elementary information processes. Some models have even differentiated between different processes (e.g., counting, multiplication, comparisons, reading) within the same experiment and estimated the duration of each of these individually. This was, however, only possible for simple models and through very clever experimental design. In contrast, estimating the duration of several interacting elementary information processes even for complex models is a straightforward extension of the EIP regression we presented in this article. Ideally, in the future EIP regression should be integrated with GOMS-like modeling frameworks, e.g. Cogulator (Estes, 2021). This would immediately make modern statistical estimation and model comparison tools available for a large class of classical cognitive models that are also widely applied in human–computer interaction.

## Figures and Tables

**Fig. 11:**
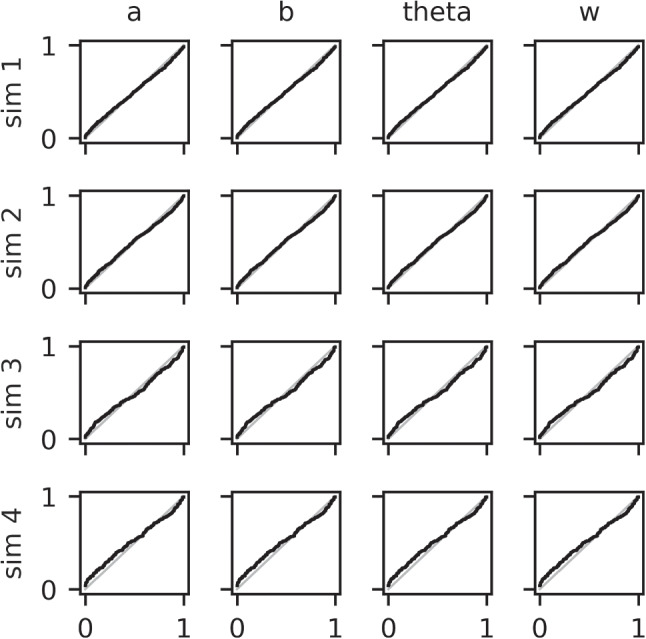
Testing whether the participant parameters are recovered well: are the quantiles distributed according to a uniform distribution? PP-plots for each parameter setting

**Fig. 12:**
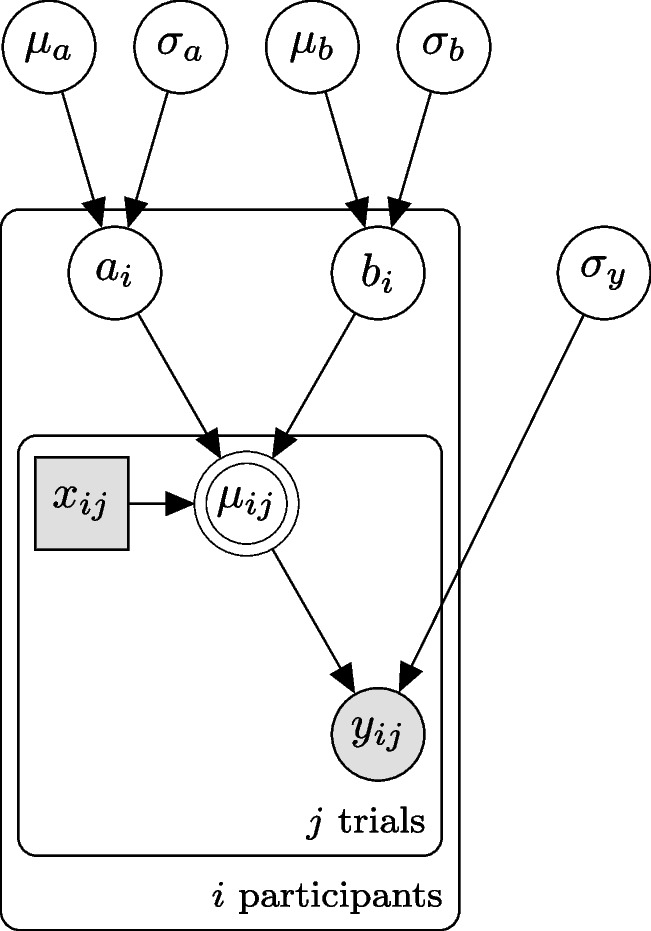
Hierarchical linear regression model for the counting trials. The predicted mean is determined by the parameters $a_i$ and $b_i$ together with the value of the min addend $x_{ij}$

**Fig. 13:**
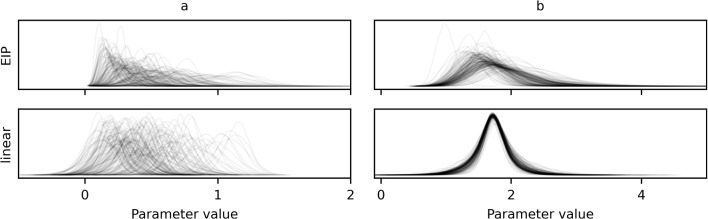
The distributions of the participant parameters for all participants for the two models of min-counting. Each *line* shows the posterior density of values for the respective variable for one participant. *Top*: EIP regression model, *bottom*: linear regression model. Note how some distributions of the variable *a* get credible values of below zero in the linear regression model

**Fig. 14:**
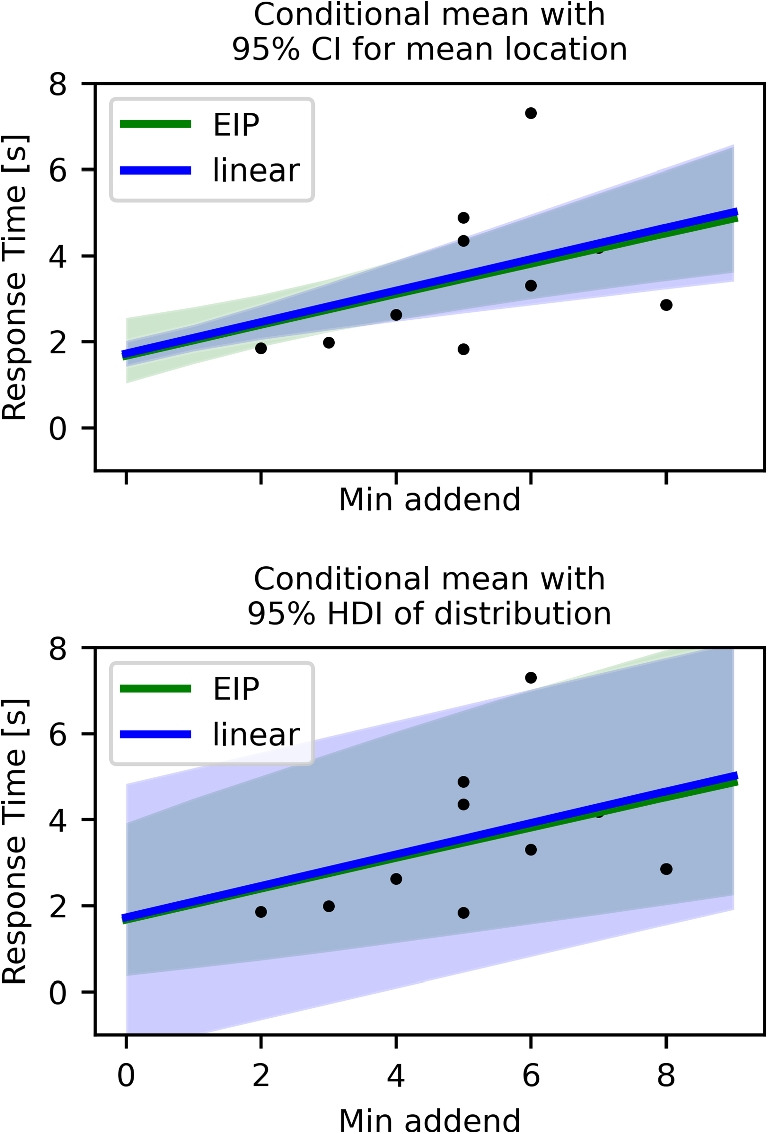
Direct comparison of the two model results. The point estimates for the regression line is very similar in both models. The biggest difference is in the lower plot. In EIP regression, the expected spread around the mean is adjusted for each participant individually. It is not symmetric around the regression line, but has a bigger spread above than below. Additionally, it increases with the value of the min addend. In the linear regression, we fit just a single spread parameter for the whole population, it is symmetric around the regression line and thus can include negative response times in the expected region

## Data Availability

The data and materials of the Sudoku study are available at https://osf.io/rgh3j/

## Appendix 1: Post hoc parameter recovery study

As a sanity check we can test how well the model fits the parameters for artificially generated data with known values. Of course, when generating data from the model, Bayesian inference should be able to re-discover the values from the generated data. Such a parameter recovery study does not tell us about the model’s adequacy for real-world data. For that aspect it is more useful to compare different models, similar to our comparison of the two Sudoku models (Lee et al., 2019, Appendix B).

### Different parameter values

When we generate data from our model we know the true values of each parameter. We can then check where the true value is in the posterior distribution. Intuitively, the best case would be if the true value is very close to the mean of the distribution. Of course, also the width of the posterior distribution should be well calibrated. More specifically, the quantiles of where the true values fall within the posterior distribution should follow a uniform distribution. Hence, when one has many true values, one can calculate for each of them the quantile within the corresponding posterior distribution of samples and check whether they are correctly distributed.

For the population parameters we used four different settings: Starting with values very similar to those found for the real data, we added a condition where the *a* parameter was bigger and the others smaller, i.e., a condition where more of the variance in the response times is explained by the process model, and two conditions where either *b* or $\theta$ was larger than in the original. Specifically, we used the following four parameter combinations for the parameters $m_a$, $m_b$, and $m_{\theta }$:

- sim 1: 1.5, 2.5, 3.5
- sim 2: 3, 1, 1
- sim 3: 1, 10, 3
- sim 4: 1, 2, 15

For the standard deviations we used 1.3, 3, 3 for $s_a$, $s_b$ and $s_{\theta }$, respectively, for all simulations. With these values set, we sampled the parameters for the individual participants as well as response times. We used the same sizes for trial and participant number as in the actual experiment described above. We sampled ten new data sets for sim 1 and sim 2 and four for the two others and refit the model on each of these data sets. We combined the quantiles for the participant parameters of the different model fits for the same parameters and show a PP-plots for all participant parameters in Fig. 11. The lines of the PP-plots are all very close to the diagonal, indicating well calibrated models. As long as the model is correctly implemented, no other result is expected when using Bayesian inference.

### Sizes of the highest density intervals with varying numbers of participants and trials

In order to quantify how many participants and trials per participant are necessary in order to reach a specific size of highest density interval (HDI), one can also simulate data of different sizes and fit the model on them. Naturally, with more data the size of the 95%-HDI shrinks. The numbers required to reach a desired size of course depend on the model specification. It thus makes sense to run such simulations with the specific model one wants to use for data analysis. To give an impression of possible results of such a study, we show different combinations of participant number and trials per participant for the experiment and model reported above (“EIP regression with strategy selection”) in Table 4. To generate the data we set the six population parameters to approximately the means found in the model for the actual experiment. We used $m_a = 1.5$, $m_b = 2.5$, $m_{\theta } = 3.5$,$s_a = 1.3$, $s_b = 3$, and $s_{\theta } = 3$.

As expected, the size of the HDI shrinks with more data. Participant parameters can be estimated more precisely with more trials per participant, whereas population level parameters get more precise estimates with an increasing number of participants. Depending on which of these values one needs to a certain precision, one can adapt the experimental design to reach the goal. One thing that becomes apparent in this simulation study is that the model has most problems in finding precise values for the *b* parameter and its priors. The sizes of the confidence intervals are much larger than for any of the other parameters.

We only fit one simulated experiment per combination of participant and trial number, due to computation time. While the smallest combination was fit in just 10 min, the largest ones needed several days on our machine, the latent steps make computations very slow. All the models are well calibrated. The true values fall evenly within the quantiles of the posterior distribution. PP-plots showing how closely the fitted distributions match the theoretical expectations can be found at https://osf.io/rgh3j/.

**Table 4:** Sizes of 95% highest density intervals for different numbers of participants and trials for the EIP regression with latent steps and strategy selection

Participants	10	10	10	50	50	50	150
Trials	30	150	450	30	150	450	30
$$m_a $$	1.92	1.94	2.42	0.68	0.47	0.49	0.32
$$m_b $$	5.57	5.61	3.66	5.97	2.90	3.44	2.52
$m_{\theta }$	4.04	3.29	3.83	1.82	1.93	2.27	1.17
$$s_a $$	3.13	2.98	4.56	0.90	0.56	0.64	0.41
$$s_b $$	56.90	101.33	75.37	53.22	16.77	17.14	17.53
$s_{\theta }$	8.83	5.54	6.76	2.67	3.12	3.99	1.90
*a*	0.59	0.36	0.16	0.86	0.34	0.23	0.67
*b*	5.58	4.29	1.32	10.27	3.95	3.42	6.58
$\theta$	3.57	1.92	1.23	4.76	2.65	1.59	4.76
*w*	0.40	0.25	0.17	0.54	0.30	0.19	0.54

Experimental design of the simulations was exactly like in the reported experiment above, only the number of participants and trials per participant were varied

## Appendix 2: Linear regression as comparison

We can also implement the traditional linear regression as a hierarchical Bayesian model. We do so to compare the results to the EIP regression model on the data of children’s addition. We estimate parameters for the mean ($a_i$) and slope ($b_i$) for each participant, the variance is a population parameter in this model. We model the participant parameters as drawn from normal distributions. The graphical model can be found in Fig. 12.

### Results linear regression

**Table 5:** The parameters found for the hierarchical linear regression. All values are in seconds

		Mean	SD	HDI 2.5%	HDI 97.5%
$\mu _a$	[s]	0.433	0.030	0.374	0.494
$\mu _b$	[s]	1.726	0.106	1.515	1.929
$\sigma _a$	[s]	0.267	0.019	0.229	0.304
$\sigma _b$	[s]	0.231	0.149	0.000	0.494
$\sigma _y$	[s]	1.584	0.028	1.533	1.643

Again, we get parameters for each participant and hyper-parameter distributions describing the group. Group parameters can be found in Table 5, the distribution of participant parameters is plotted in Fig. 13. The regression line for a single, exemplary participant can be seen in Fig. 14.

### Comparing the results of the two models

The two models, EIP regression and standard linear regression, use different distributions for the priors as well as the spread around the predicted mean, so one would expect slightly different results. An important difference is also that the EIP regression model fits a variance parameter for each individual participant, whereas in the linear regression model the variance parameter is shared by all participants. However, generally the two models agree very well with each other. In the group parameters, the means and standard deviations of *a* and *b* are almost identical, only the standard deviation for the *b* parameter is bigger in the EIP regression model than the linear regression. The parameters per participant can be compared easily. $a_i$ is the value of the slope, $b_i$ the value of the intercept for each participant. Both models try to fit the mean of the participant data. In Fig. 13 the densities of all participants for both parameters are depicted. The rough ranges of plausible parameters is the same in both models, in the linear regression model some values below zero are included, which is excluded by the specification of the EIP regression model.

It is also possible to translate the parameters into regression lines for single participants in both models. An example can be seen in Fig. 14. The mean prediction is very similar in both models. The biggest difference between the two models is how the variance of the data points around the mean is handled. In the linear regression model, steps of the same duration are added and variance is only added via a normal distribution around the so calculated mean in the end. In the EIP regression model on the other hand, we assume that each step that contributes to the overall time is a random variable that can have different duration. So a larger spread for bigger values of the min addend ($x_{ij}$) is expected by the EIP regression model but not by the linear regression model. In the linear regression model, the standard deviation of the normal distribution is just one parameter that is shared by all participants, in the EIP regression model the precision parameter $\theta _i$ is fit for each participant *i* and can thus be different across participants. An obvious advantage of the Gamma density is, that it will never place probability mass below zero. Statistically, using the estimated leave-one-out ELPD or cross validation ELPD, the EIP regression model has a much better log-likelihood ($-2783.81$ with standard error 49.85 for the EIP model and $-3473.51$ with standard error 115.58, the difference is thus 689.70 with a standard error of 91.34). Hence, while the EIP regression is theoretically clearly superior, in this example, it does lead to conclusions that are extremely similar to those of a standard linear regression analysis – as performed in the study of Hopkins and Bayliss Hopkins & Bayliss (2017), whose data we analyzed here.

## Footnotes

1 Some differences in EIP step counts arise due to the order in which numbers are tried. If a smaller number is tried first and it is the required target digit, then this puzzle has a lower number of EIP steps, but we do not want to make the strong assumption that our participants always follow this exact order. So we lump all target number scan distributions together.

2 For some models it will be easy to compute this distribution by hand, but in general it will be easier to estimate this distribution by sampling.

## Code availability

The analysis code is available at https://osf.io/rgh3j/
